# Supplementary material for: Utilising multi-modal data-driven network analysis to identify monotherapy and combinational therapy targets in SOX2-dependent squamous cell lung cancer
Source: Commun Chem. 2025 Dec 12;8:401. doi: 10.1038/s42004-025-01778-7 (PMC12717043; doi:10.1038/s42004-025-01778-7)
Supplement: Supplementary file 2 — Supplementary Information [file 42004_2025_1778_MOESM2_ESM.pdf]

# Utilising multi-modal data-driven network analysis to identify monotherapy and combinational therapy targets in SOX2-dependent squamous cell lung cancer

Woochang Hwang[1,2*‡], Daniel Kottmann[3*‡], Wenrui Guo[3], Méabh MacMahon[1,2,4], Lucia Correia[3‡], Sherine Ahmed[3], Rebecca Harris[1‡], Frank McCaughan[3†], Namshik Han[1,5,6,7,8,9†]

[1]Milner Therapeutics Institute, University of Cambridge, Cambridge, UK

[2]CardiaTec Biosciences LTD, Cambridge, UK

[3]Victor Phillip Dahdaleh Heart and Lung Research Institute, Department of Medicine, University of Cambridge, Cambridge, UK

[4]Centre for Therapeutics Discovery, LifeArc, Stevenage, UK

[5]Cambridge Centre for AI in Medicine, Department of Applied Mathematics and Theoretical Physics, University of Cambridge, Cambridge, UK

[6]Cambridge Stem Cell Institute, University of Cambridge, Cambridge, UK

[7]Department of Quantum Information, Institute for Convergence Research and Education in Advanced Technology and Engineering, Yonsei University, Seoul, Republic of Korea

[8]Department of Nano Biomedical Engineering (NanoBME), Advanced Science Institute, Yonsei University, Seoul, Republic of Korea

[9]Center for Nanomedicine, Institute for Basic Science (IBS), Seoul, Republic of Korea

[*]These authors contributed equally.

[†]These authors jointly supervised this work.

[‡]The current address: WH and MM - CardiaTec Biosciences, Cambridge, UK; DK – Boston Consulting Group, Berlin, Germany; LC - Adaptimmune, Abingdon, UK; RH - Cancer Research Horizons, Cambridge, UK

Correspondence: fm319@cam.ac.uk, nh417@cam.ac.uk

The PDF file includes:

Supplementary Figure 1 to Supplementary Figure 7

Supplementary Table 1 to Supplementary Table 6

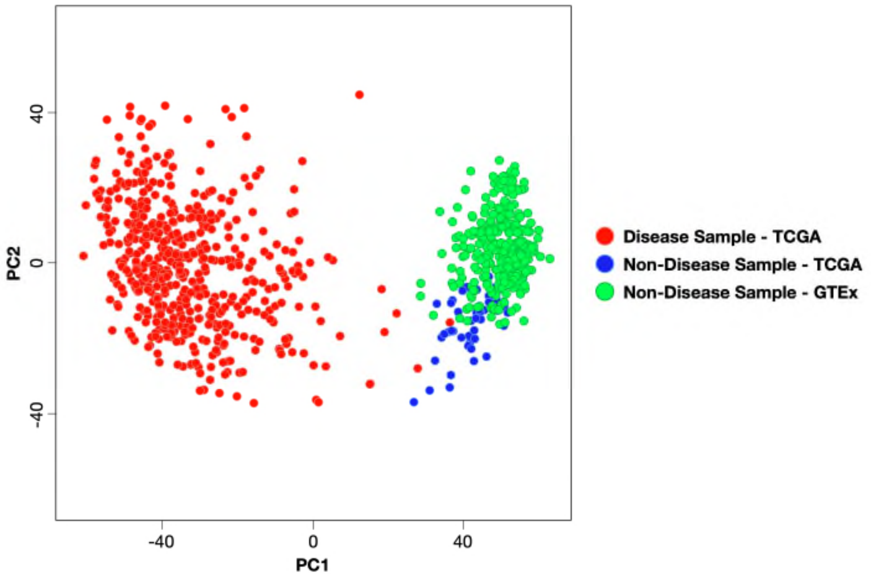


**Supplementary Figure 1: Principal Component Analysis of Disease and Non-Disease Samples in TCGA and GTEx**

Principal Component Analysis (PCA) of RNA-seq data from disease (TCGA) and non-disease samples (TCGA and GTEx). Each point represents a sample, with color denoting the sample type: disease samples from TCGA (red), non-disease samples from TCGA (blue), and non-disease samples from GTEx (green). PCA was performed on normalized expression profiles, illustrating the separation between disease and non-disease states.

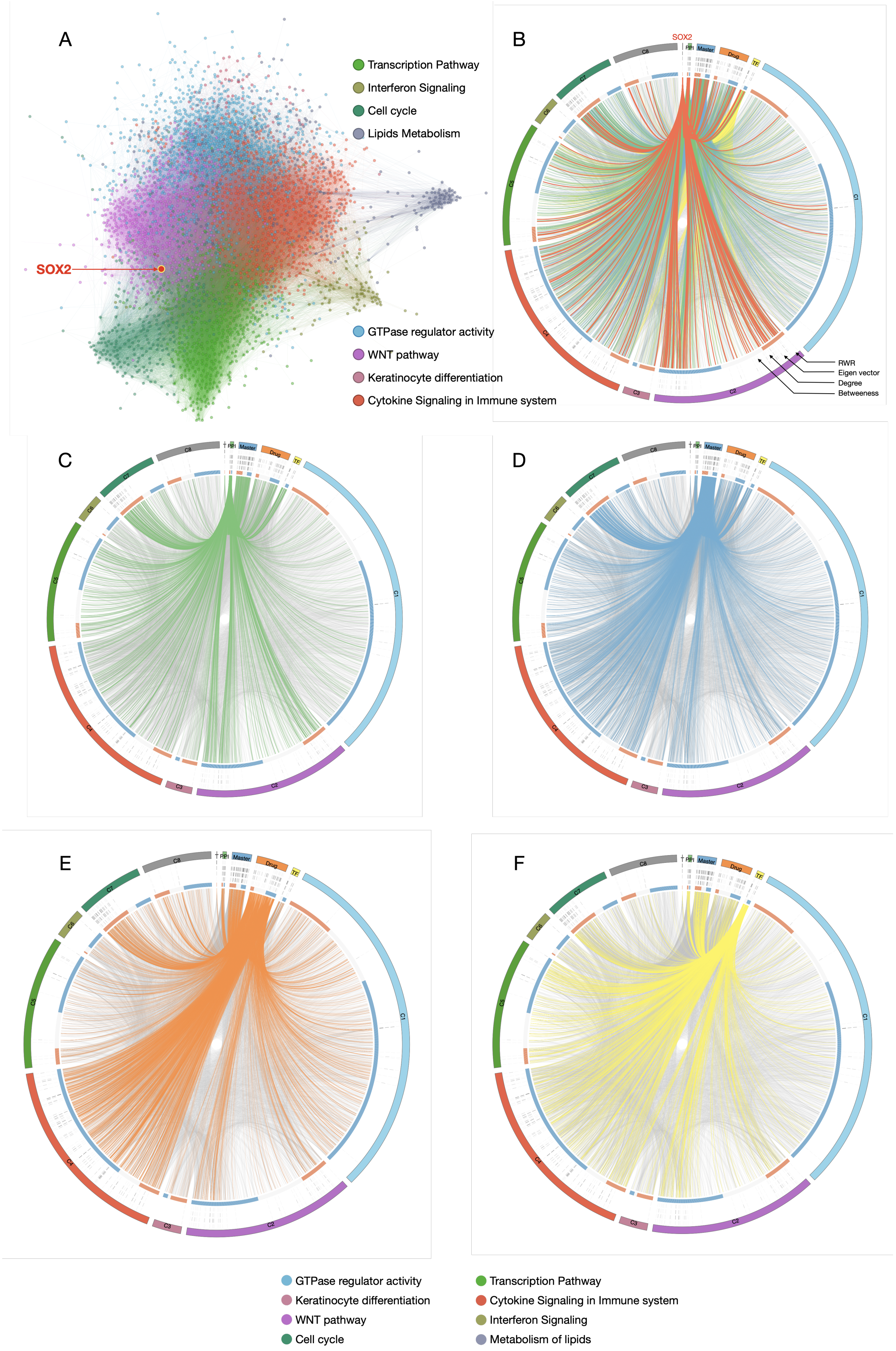
A
Transcription Pathway
Interferon Signaling
Cell cycle
Lipids Metabolism
SOX2
GTPase regulator activity
WNT pathway
Keratinocyte differentiation
Cytokine Signaling in Immune system
B
SOX2
PPI
Master
Drug
TF
C1
C2
C3
C4
C5
C6
C7
C8
RWR
Eigen vector
Degree
Betweeness
C
D
E
F
GTPase regulator activity
Keratinocyte differentiation
WNT pathway
Cell cycle
Transcription Pathway
Cytokine Signaling in Immune system
Interferon Signaling
Metabolism of lipids

**Supplementary Figure 2: Circos plots of LUSOX**

**A)** The SOX2-dependent network consists of 2,829 genes and 39,394 interactions. The network is divided into eight communities, each associated with key pathways, including the Cell Cycle, Cytokine Signaling, Interferon Signaling, Lipid Metabolism, RHO GTPase Cycle, and WNT Pathway. Transcription Pathway refers to the community where key transcription factors and their target genes cluster, reflecting their central regulatory role.
**B)** Circos plot of the SOX2-dependent network showing protein interactions (inner links), fold change values in LUSC (outermost track: red indicates upregulation, blue indicates downregulation), and network centrality scores (RWR, Eigenvector, Degree, and Betweenness centralities). The outer band colors represent pathway communities, consistent with panel A. SOX2 interacts directly with master regulators of LUSC, including transcription factors and proteins in cytokine signaling and WNT pathways. These master regulators bridge SOX2 to the broader network of 2,829 proteins, underscoring SOX2's influence across pathways. Proteins associated with LUSC drugs represent targets from clinical or preclinical studies for lung squamous cell carcinoma, and the cytokine signaling pathway contains the highest number of core proteins.

**C)** Highlight of interactions from SOX2 PPI

**D)** Highlight of interactions from Master proteins of LUSC

**E)** Highlight of interactions from LUSC drugs

**F)** Highlight of interactions from TF signatures

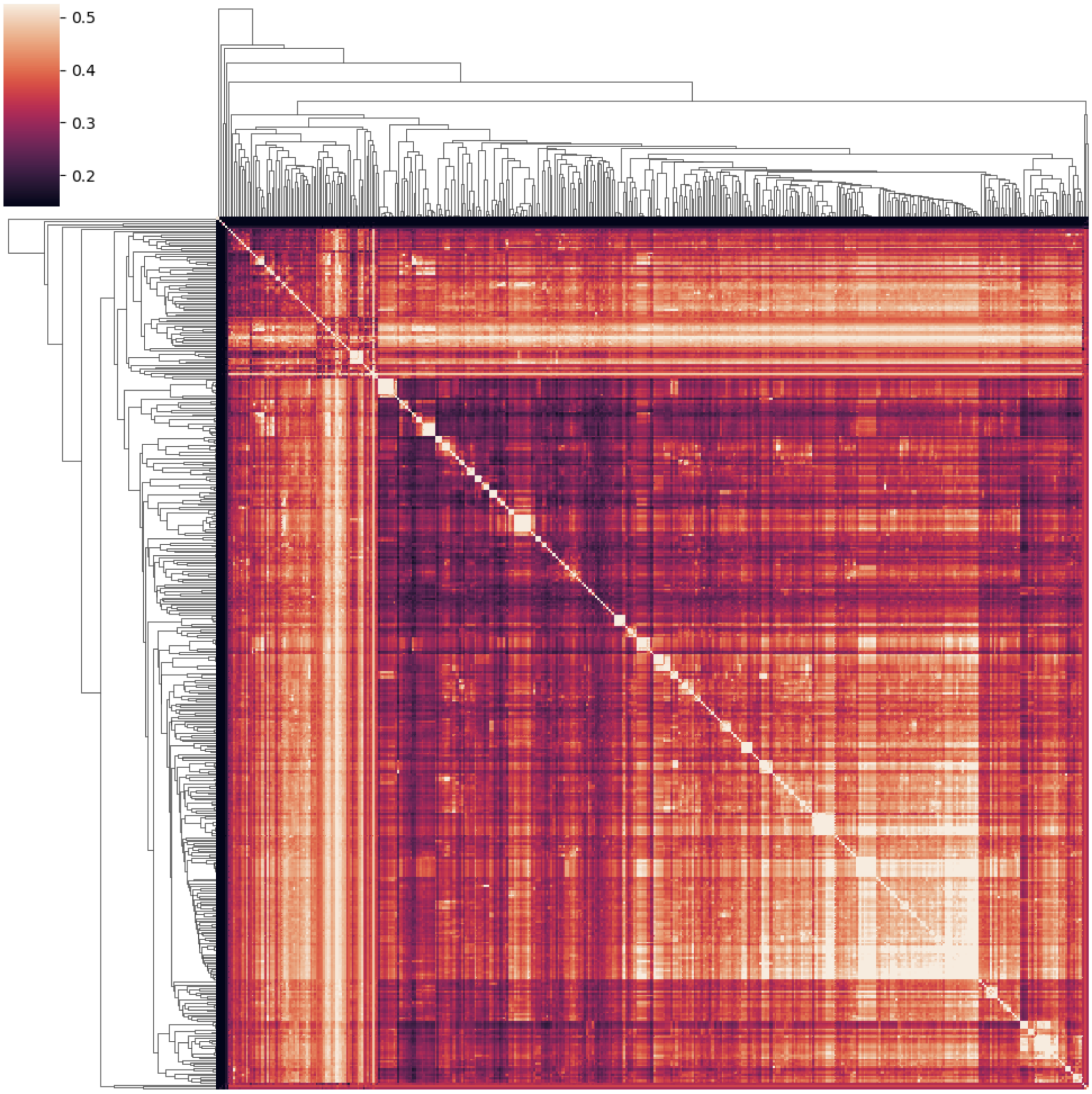


**Supplementary Figure 3: Structure-based clustering of candidate drugs.**

Cluster heatmap of candidate drugs, grouped by chemical structure. Brighter colours indicate higher structural similarity, while white blocks along the diagonal represent clusters of drugs that share structural and functional characteristics.

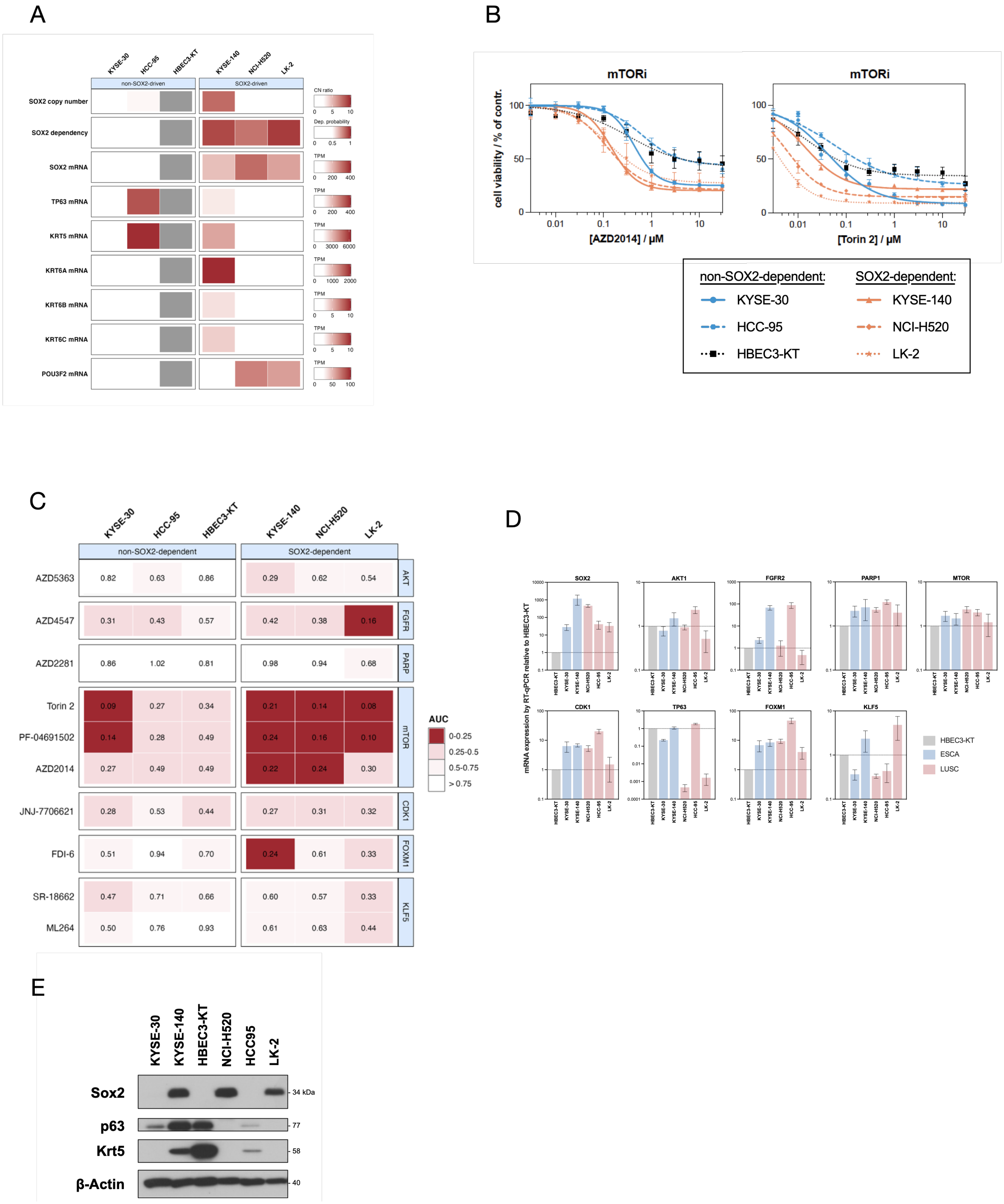
A
KYSE-30
HCC-95
HBEC3-KT
KYSE-140
NCI-H520
LK-2
non-SOX2-driven
SOX2-driven
SOX2 copy number
SOX2 dependency
SOX2 mRNA
TP63 mRNA
KRT5 mRNA
KRT6A mRNA
KRT6B mRNA
KRT6C mRNA
POU3F2 mRNA
CN ratio
Dep. probability
TPM
B
mTORi
cell viability / % of contr.
[AZD2014] / µM
[Torin 2] / µM
non-SOX2-dependent:
KYSE-30
HCC-95
HBEC3-KT
SOX2-dependent:
KYSE-140
NCI-H520
LK-2
C
non-SOX2-dependent
SOX2-dependent
AZD5363 0.82 0.63 0.86 0.29 0.62 0.54 AKT
AZD4547 0.31 0.43 0.57 0.42 0.38 0.16 FGFR
AZD2281 0.86 1.02 0.81 0.98 0.94 0.68 PARP
Torin 2 0.09 0.27 0.34 0.21 0.14 0.08
PF-04691502 0.14 0.28 0.49 0.24 0.16 0.10 mTOR
AZD2014 0.27 0.49 0.49 0.22 0.24 0.30
JNJ-7706621 0.28 0.53 0.44 0.27 0.31 0.32 CDK1
FDI-6 0.51 0.94 0.70 0.24 0.61 0.33 FOXM1
SR-18662 0.47 0.71 0.66 0.60 0.57 0.33
ML264 0.50 0.76 0.93 0.61 0.63 0.44 KLF5
AUC
0-0.25
0.25-0.5
0.5-0.75
> 0.75
D
mRNA expression by RT-qPCR relative to HBEC3-KT
SOX2
AKT1
FGFR2
PARP1
MTOR
CDK1
TP63
FOXM1
KLF5
HBEC3-KT
ESCA
LUSC
E
KYSE-30
KYSE-140
HBEC3-KT
NCI-H520
HCC95
LK-2
Sox2
p63
Krt5
β-Actin
34 kDa
77
58
40

**Supplementary Figure 4: Target validation for monotherapy**

**A**) Characterisation of cell lines with SOX2 copy number (CN) ratio (1 means same CN as ploidy), SOX2 dependency probability (higher means more dependent), and mRNA expression of squamous markers. Data from the CCLE and DepMap projects.

**B)** Dose–response profiles of multiple mTOR inhibitors tested in parallel across the same panel of cell lines.

**C)** Heatmap showing drug sensitivity in SOX2-dependent and non-SOX2-dependent cell lines by area under the curve (AUC) values calculated from dose-response curves (see Figure 3E).

**D)** Bar charts showing the mRNA expression of target genes in the cell line panel by RT-qPCR. Gene expression was normalised to TBP and is shown relative to HBEC3-KT. Data presented as mean ± SEM of N = 3 biological replicates (n = 3 technical replicates).

**E)** Western blot showing the expression of selected squamous markers in the cell line panel.

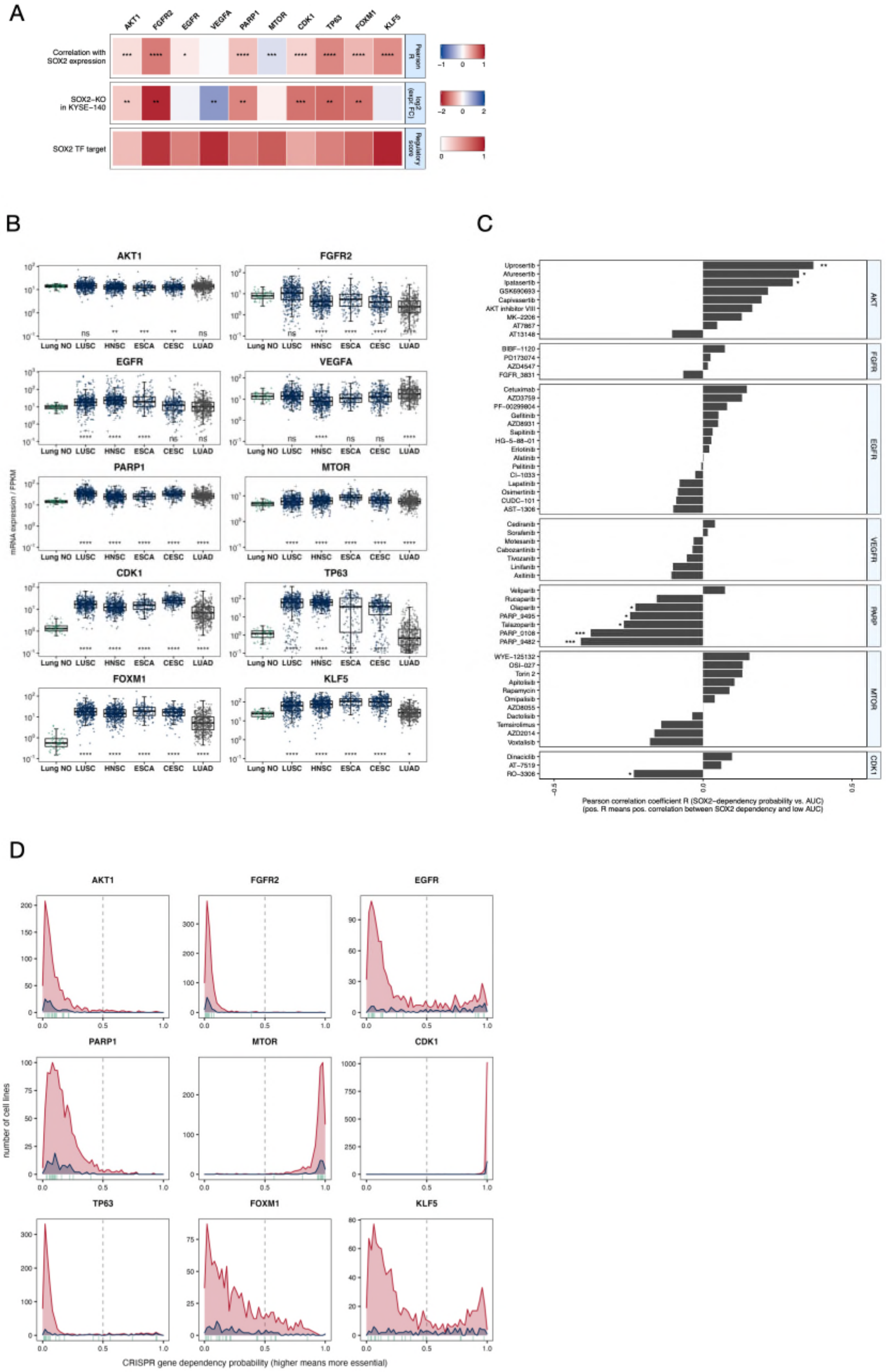
A
AKT1
FGFR2
EGFR
VEGFA
PARP1
MTOR
CDK1
TP63
FOXM1
KLF5
Correlation with SOX2 expression
SOX2–KO in KYSE–140
SOX2 TF target
Pearson R
log2 (expr. FC)
Regulatory score
B
C
D
mRNA expression / FPKM
Lung NO
LUSC
HNSC
ESCA
CESC
LUAD
Pearson correlation coefficient R (SOX2–dependency probability vs. AUC)
(pos. R means pos. correlation between SOX2 dependency and low AUC)
AKT
FGFR
EGFR
VEGFR
PARP
MTOR
CDK1
number of cell lines
CRISPR gene dependency probability (higher means more essential)

**Supplementary Figure 5: Targets for monotherapy**

**A**) Relationship between SOX2 and prioritised targets

Pearson correlation coefficient R for target gene vs. SOX2 mRNA expression with significance of correlation. Data from the TCGA LUSC project. Higher value (red) means higher correlation of target expression with SOX2 expression. mRNA expression fold change in KYSE-140 cells after SOX2 knockout. Significance from t-test comparing SOX2 knockout with control. Data from Liu et al. Higher value (red) means overexpressed after SOX2 knockout. Regulatory potential score for the SOX2 TF to regulate the target genes. Higher score (red) means higher regulatory potential of target gene by SOX2.

**B)** Expression of prioritised targets in squamous tumours, LUAD and normal lung tissue in TCGA datasets.

**C)** The correlation between SOX2 dependency and drug sensitivity, measured by area under the curve (AUC), was analysed for drugs targeting the prioritised targets identified from the iPANDDA pipeline. This analysis utilised drug sensitivity data from the Genomics of Drug Sensitivity in Cancer (GDSC) project across 77 squamous cancer cell lines. Pearson correlation coefficients between SOX2 dependency and drug sensitivity AUC values were calculated, along with their statistical significance.

**D)** Dependency of target candidates in cell lines: Density plots showing the number of cell lines with target gene dependency probabilities. Higher gene dependency probability value means the gene is more essential in a cell line. All screened cell lines (n = 1086) are shown in red, squamous cell lines (n = 119) are shown in blue, and LUSC cell lines (n = 22) are highlighted with green stripes.

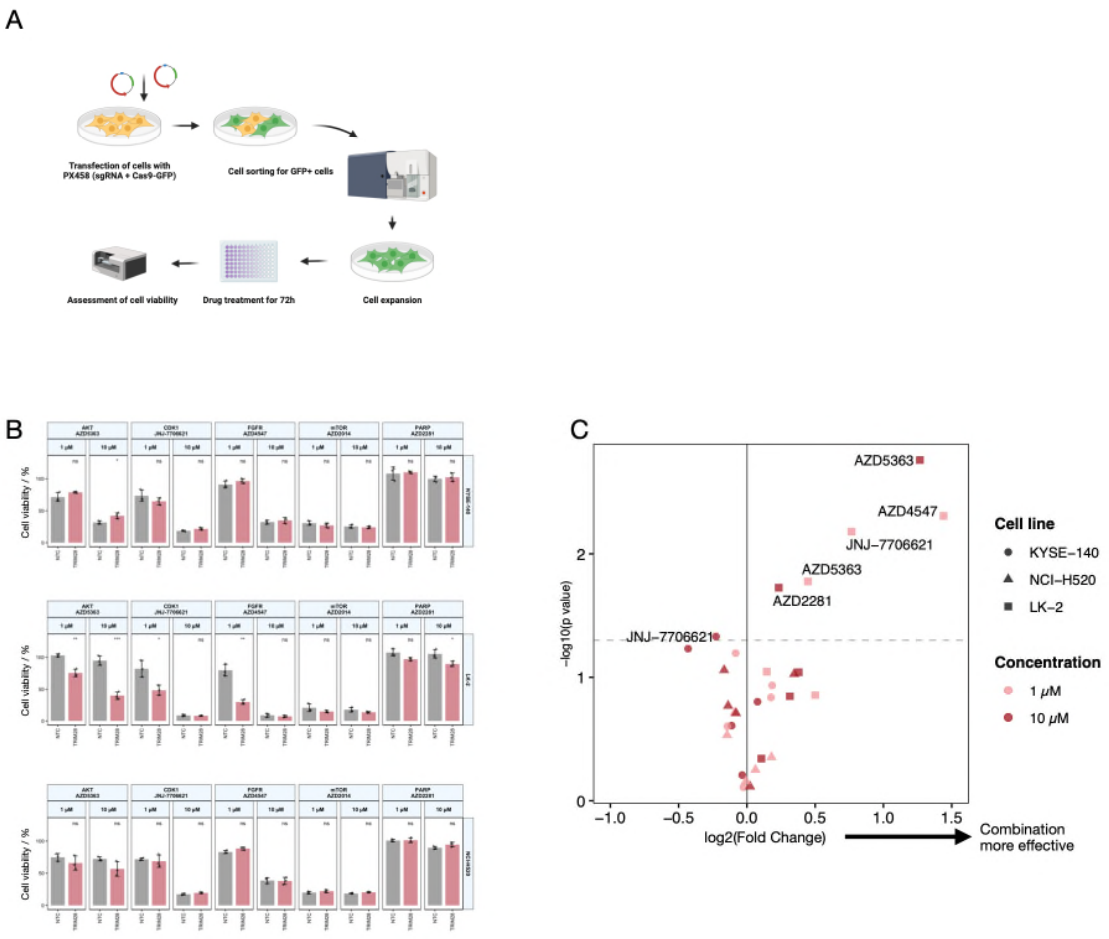


**Supplementary Figure 6: Target validation for combination therapy**

**A**) Schematic overview of the experimental design to evaluate combination therapies.

**B)** Individual plots of cell viability after treatment of cell lines with selected compounds in combination with sgNTC or sgTRIM28. CRISPR/Cas9-engineered cells were expanded and then treated with 1 µM or 10 µM of selected compounds for 72h and cell viability assessed. Data presented as means ± SEM of N = 3 biological replicates (n = 3) technical replicates.

C) **Full list of drugs used in the combination treatment with TRIM28 knockout.**

This table lists all drugs included in the volcano plot shown in **Figure 5C**, targeting AKT, CDK1, FGFR, mTOR, and PARP pathways. The data correspond to differential effects observed upon TRIM28 knockout, as visualised in the main figure.

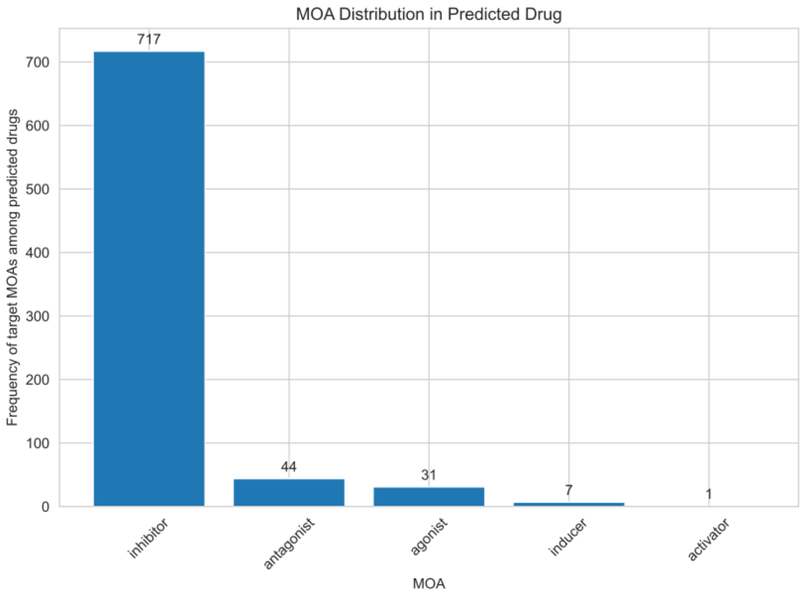


**Supplementary Figure 7: Distribution of mechanisms of action (MOAs) among predicted drug candidates.**

Bar plot illustrating the frequency of annotated MOAs for compounds identified through in silico drug simulations. The majority of compounds (>90%) are classified as **inhibitors**, with smaller proportions annotated as antagonists, agonists, inducers, or activators. This distribution informed our decision to exclude targets with decreased expression in LUSC, as their inhibition would be biologically inconsistent with therapeutic intent.

(Table in the Supplementary Data 1.xlsx file)

**Supplementary Table 1**: Multi-modal data integration result table

| **Index** | **Name** | **P-value** | **Adjusted p-value** |
|---|---|---|---|
| 1 | Lung | 1.16E-47 | 9.72E-46 |

**Supplementary Table 2** the Human Protein Atlas enrichment test for LUSC specific genes from TCGA ANOVA test.

(Table in the Supplementary Data 2 .xlsx file)

**Supplementary Table 3**: Network analysis result table

(Table in the Supplementary Data 3 .xlsx file)

**Supplementary Table 4**: Drug simulation result table

(Table in the Supplementary Data 4 .xlsx file)

**Supplementary Table 5**: Drug cluster table

(Table in the Supplementary Data 5 .xlsx file)

**Supplementary Table 6**: Target prioritisation results table: List of prioritised target candidates for SOX2-driven LUSC with their implication in LUSC and other squamous cancers and described link with SOX2
