# Supplementary material for: Utilising multi-modal data-driven network analysis to identify monotherapy and combinational therapy targets in SOX2-dependent squamous cell lung cancer
Source: Commun Chem. 2025 Dec 12;8:401. doi: 10.1038/s42004-025-01778-7 (PMC12717043; doi:10.1038/s42004-025-01778-7)
Supplement: Supplementary file 3 — Description of Supplementary Data [file 42004_2025_1778_MOESM3_ESM.pdf]

Description of Supplementary Data

File Name : Supplementary Data1.xlsx file

Description: Supplementary Table 1: Multi-modal data integration result table

File Name : Supplementary Data2.xlsx file

Description: Supplementary Table 3: Network analysis result table

File Name : Supplementary Data3.xlsx file

Description: Supplementary Table 4: Drug simulation result table

File Name : Supplementary Data4 .xlsx file

Description: Supplementary Table 5: Drug cluster table

File Name : Supplementary Data5.xlsx file

Description: Supplementary Table 6: Target prioritisation results table: List of prioritised target candidates for SOX2-driven LUSC with their implication in LUSC and other squamous cancers and described link with SOX2
