# Supplementary material for: Utilising multi-modal data-driven network analysis to identify monotherapy and combinational therapy targets in SOX2-dependent squamous cell lung cancer
Source: Commun Chem. 2025 Dec 12;8:401. doi: 10.1038/s42004-025-01778-7 (PMC12717043; doi:10.1038/s42004-025-01778-7)
Supplement: Supplementary file 9 — nr-reporting-summary [file 42004_2025_1778_MOESM9_ESM.pdf]

nature portfolio

Corresponding author(s): Namshik Han, Frank McCaughan

Last updated by author(s): Sep 17, 2025

Reporting Summary

Nature Portfolio wishes to improve the reproducibility of the work that we publish. This form provides structure for consistency and transparency in reporting. For further information on Nature Portfolio policies, see our Editorial Policies and the Editorial Policy Checklist.

Statistics

For all statistical analyses, confirm that the following items are present in the figure legend, table legend, main text, or Methods section.

n/a	Confirmed	
☐	☒	The exact sample size (*n*) for each experimental group/condition, given as a discrete number and unit of measurement
☐	☒	A statement on whether measurements were taken from distinct samples or whether the same sample was measured repeatedly
☐	☒	The statistical test(s) used AND whether they are one- or two-sided<br>*Only common tests should be described solely by name; describe more complex techniques in the Methods section.*
☐	☒	A description of all covariates tested
☐	☒	A description of any assumptions or corrections, such as tests of normality and adjustment for multiple comparisons
☐	☒	A full description of the statistical parameters including central tendency (e.g. means) or other basic estimates (e.g. regression coefficient) AND variation (e.g. standard deviation) or associated estimates of uncertainty (e.g. confidence intervals)
☐	☒	For null hypothesis testing, the test statistic (e.g. *F*, *t*, *r*) with confidence intervals, effect sizes, degrees of freedom and *P* value noted<br>*Give P values as exact values whenever suitable.*
☐	☒	For Bayesian analysis, information on the choice of priors and Markov chain Monte Carlo settings
☐	☒	For hierarchical and complex designs, identification of the appropriate level for tests and full reporting of outcomes
☐	☒	Estimates of effect sizes (e.g. Cohen's *d*, Pearson's *r*), indicating how they were calculated

Our web collection on statistics for biologists contains articles on many of the points above.

Software and code

Policy information about availability of computer code

Data collection	The LUSC-TCGA TARGET GTEx dataset, STRING v11.5, OpenTargets 19.09, DrugBank(v5.1), STITCH(v5.0), DepMap (Broad v21Q1)
Data analysis	All code is written in Python or R. It requires pandas>=1.3.0, numpy>=1.23.0, scipy>=1.10.0, networkx>=2.2 matplotlib>=3.6.0, seaborn>=0.12.0. All code is at GitHub (https://github.com/Namshik-Han-Lab/iPANDDA.git) and Zenodo repository((https://doi.org/10.5281/zenodo.17143330).

For manuscripts utilizing custom algorithms or software that are central to the research but not yet described in published literature, software must be made available to editors and reviewers. We strongly encourage code deposition in a community repository (e.g. GitHub). See the Nature Portfolio guidelines for submitting code & software for further information.

Data

Policy information about availability of data

All manuscripts must include a data availability statement. This statement should provide the following information, where applicable:

- Accession codes, unique identifiers, or web links for publicly available datasets
- A description of any restrictions on data availability
- For clinical datasets or third party data, please ensure that the statement adheres to our policy

The datasets generated during and/or analysed during the current study are available in the Zenodo repository (https://doi.org/10.5281/zenodo.17143330).

Research involving human participants, their data, or biological material

Policy information about studies with human participants or human data. See also policy information about sex, gender (identity/presentation), and sexual orientation and race, ethnicity and racism.

Reporting on sex and gender	This study did not involve human participants, human data, or human biological materials.
Reporting on race, ethnicity, or other socially relevant groupings	This study did not involve human participants, human data, or human biological materials.
Population characteristics	This study did not involve human participants, human data, or human biological materials.
Recruitment	This study did not involve human participants, human data, or human biological materials.
Ethics oversight	This study did not involve human participants, human data, or human biological materials.

Note that full information on the approval of the study protocol must also be provided in the manuscript.

Field-specific reporting

Please select the one below that is the best fit for your research. If you are not sure, read the appropriate sections before making your selection.

☒ Life sciences ☐ Behavioural & social sciences ☐ Ecological, evolutionary & environmental sciences

For a reference copy of the document with all sections, see nature.com/documents/nr-reporting-summary-flat.pdf

Life sciences study design

All studies must disclose on these points even when the disclosure is negative.

Sample size	Sample size was determined based on previous publications and consistency across biological replicates.
Data exclusions	No data were excluded unless technical failure was confirmed.
Replication	Each experiment was replicated three times independently with consistent results.
Randomization	No randomization was used, as group allocation was determined by treatment conditions.
Blinding	Blinding was also not performed, as the control and treated conditions were clearly distinguished.

Reporting for specific materials, systems and methods

We require information from authors about some types of materials, experimental systems and methods used in many studies. Here, indicate whether each material, system or method listed is relevant to your study. If you are not sure if a list item applies to your research, read the appropriate section before selecting a response.

Materials & experimental systems

n/a	Involved in the study	
☒	☐	Antibodies
☒	☐	Eukaryotic cell lines
☒	☐	Palaeontology and archaeology
☒	☐	Animals and other organisms
☒	☐	Clinical data
☒	☐	Dual use research of concern
☒	☐	Plants

Methods

n/a	Involved in the study	
☐	☒	ChIP-seq
☒	☐	Flow cytometry
☒	☐	MRI-based neuroimaging

Plants

Seed stocks	*Report on the source of all seed stocks or other plant material used. If applicable, state the seed stock centre and catalogue number. If plant specimens were collected from the field, describe the collection location, date and sampling procedures.*
Novel plant genotypes	*Describe the methods by which all novel plant genotypes were produced. This includes those generated by transgenic approaches, gene editing, chemical/radiation-based mutagenesis and hybridization. For transgenic lines, describe the transformation method, the number of independent lines analyzed and the generation upon which experiments were performed. For gene-edited lines, describe the editor used, the endogenous sequence targeted for editing, the targeting guide RNA sequence (if applicable) and how the editor was applied.*
Authentication	*Describe any authentication procedures for each seed stock used or novel genotype generated. Describe any experiments used to assess the effect of a mutation and, where applicable, how potential secondary effects (e.g. second site T-DNA insertions, mosiacism, off-target gene editing) were examined.*

ChIP-seq

Data deposition

☒ Confirm that both raw and final processed data have been deposited in a public database such as GEO.

☒ Confirm that you have deposited or provided access to graph files (e.g. BED files) for the called peaks.

Data access links<br>*May remain private before publication.*	https://doi.org/10.5281/zenodo.17143330
Files in database submission	Elute-Dox-D12-merged.bw, Elute-Dox-D16-merged.bw, Elute-No-Dox-D12-merged.bw, Elute-No-Dox-D16-merged.bw, Input-Dox-D12-merged.bw, Input-Dox-D12-merged.bw, Input-No-Dox-D12-merged.bw, Input-No-Dox-D16-merged.bw
Genome browser session (e.g. UCSC)	No longer applicable

Methodology

Replicates	Pooling: 5 transwells per condition were pooled to generate sufficient cell number.
Sequencing depth	20M
Antibodies	Goat anti-SOX2 (AF2018) – target antibody. Goat anti-IgG (AB-108) – negative control antibody.
Peak calling parameters	MACS2
Data quality	High-quality reads were aligned to the human reference genome (GRCh38) using Bowtie2
Software	FastQC, Bowtie2, Samtools, Picard Tools, deepTools (bamCoverage, bamCompare)
